# Improving bedside airway tests accuracy for predicting difficult laryngoscopy using ultrasound-measured skin-to-epiglottis distance

**DOI:** 10.1186/s44158-026-00426-3

**Published:** 2026-06-17

**Authors:** Haytham Eltrabily, Maha Mostafa, Esraa Gamal Hamad, Ahmed Hasanin, Sherin Refaat, Mohamed Mansour, Sherif Alaa Embaby, Mostafa Nagy

**Affiliations:** Department of Anesthesia and Critical Care Medicine, Faculty of Medicine, Cairo University, Cairo, Egypt

**Keywords:** Airway management, Laryngoscopy, Difficult intubation, Ultrasonography, Predictive value of tests

## Abstract

**Background:**

This study aimed to assess the accuracy of a composite score combining ultrasound-measured skin-to-epiglottis distance (DSE), Upper Lip Bite Test (ULBT), and Modified Mallampati Test (MMT) in predicting difficult airway.

**Methods:**

In this prospective observational study, 250 adults scheduled for elective surgery were assessed preoperatively using ULBT, MMT, and ultrasound measurement of DSE. A composite airway score was calculated by assigning one point for each of the following: DSE > 2.0 cm, ULBT class > I, and MMT grade > II. Difficult laryngoscopy was defined as Cormack–Lehane grade > 2, and difficult intubation as an Intubation Difficulty Scale > 5. The outcomes were the ability of the score and individual tests to predict difficult airway using area under the receiver operating characteristic curve (AUC).

**Results:**

The incidence of difficult laryngoscopy and intubation was 12.8% and 6.8%, respectively. The composite score showed superior predictive accuracy compared to individual tests, with AUCs (95% confidence interval) of 0.77 (0.72–0.82) for difficult laryngoscopy and 0.83 (0.78–0.88) for difficult intubation. A score > 1 had negative predictive values of 93% for difficult laryngoscopy and 97% for difficult intubation. When all three tests were negative, the composite score had a sensitivity and negative predictive value of 100% for predicting difficult intubation.

**Conclusion:**

A composite airway score combining DSE, ULBT, and MMT can accurately predict difficult laryngoscopy and intubation. When all three parameters are negative, difficult intubation is very unlikely. Additionally, a score of < 2 rules out difficult laryngoscopy and intubation with 93% and 97% accuracy, respectively.

## Introduction

Endotracheal intubation is an essential intervention in anesthesia, emergency and critical care medicine practice which enables securing unprotected airway and allows lung ventilation. However, difficult intubation remains a major challenge which sometimes progresses to a life-threatening situation. Early identification of patients at risk of difficult intubation enables clinicians to prepare alternative or advanced airway management strategies, thereby improving safety and outcomes [1].

Several bedside clinical tests are currently used to assess airway difficulty. Among the most common are the Upper Lip Bite Test (ULBT) and the Modified Mallampati Test (MMT). According to a large meta-analysis evaluating the diagnostic performance of bedside airway tests, ULBT and MMT demonstrated the highest sensitivity for predicting difficult laryngoscopy and intubation, respectively. However, their pooled sensitivities remain modest—67% for ULBT and 51% for MMT—limiting their utility as standalone screening tools [2]. As such, current guidelines recommend a multifactorial approach to airway assessment, incorporating multiple clinical predictors [1].

In recent years, ultrasound has gained attention as a non-invasive, point-of-care tool for airway assessment. Among the sonographic parameters, the distance from skin-to-epiglottis (DSE) has shown the most consistent predictive performance, with a reported sensitivity of up to 80% [3, 4].

Some studies have shown that combining clinical and ultrasound variables results in better prediction of difficult airways than relying on single parameters [5, 6]. Therefore, we hypothesize that combining DSE with traditional clinical tests such as ULBT and MMT may enhance the overall predictive value and accuracy of airway assessments.

This study aimed to assess the accuracy of a composite score combining DSE, ULBT, and MMT in predicting difficult airway in adults undergoing elective surgery under general anesthesia.

## Patients and methods

This prospective observational study was conducted at Cairo University Hospital from June 2022 to May 2024, after institutional Research Ethics Committee approval (MS-122–2022). Written informed consent was obtained from all patients before enrollment.

Participants were adult patients (above > 20 years) with American Society of Anesthesiologists physical status (ASA) I–III scheduled for elective surgery under general anesthesia with endotracheal intubation using direct laryngoscopy.

Patients were excluded if they had a history of difficult intubation or any airway abnormalities that require an alternative technique other than direct laryngoscopy. Also, edentulous patients, patients undergoing emergency procedure, patients with body mass index > 35 kg/m^2^, and pregnant women were excluded from the study.

In the pre-anesthetic room, intravenous access was established, and a comprehensive airway evaluation was conducted by an experienced anesthetist (with more than 3 years of experience) who also managed the patient’s airway but was blinded to the ultrasound findings.

The ULBT was performed in the sitting position. Patients were instructed to bite their upper lip with their lower incisors. Classification was as follows: Class I: “Lower incisors could bite above the vermilion line”; Class II: “Bite was below the vermilion line”; Class III: “Unable to bite the upper lip” [7].

The MMT was conducted with the patient sitting and head in a neutral position. Patients were asked to open their mouth widely and protrude the tongue without phonation. Oropharyngeal visibility was graded as grade I: if soft palate, uvula, and tonsillar pillars were fully visible; grade II: if soft palate, tonsillar pillars, and uvula were partially visible; grade III: only soft palate was visible; grade IV: only hard palate was visible [8].

Additional bedside airway assessments included mouth opening, thyromental distance, sternomental distance (measured with a ruler), and neck mobility. Demographic data (age, sex, BMI) and ASA physical status were also recorded.

**Fig. 1:**
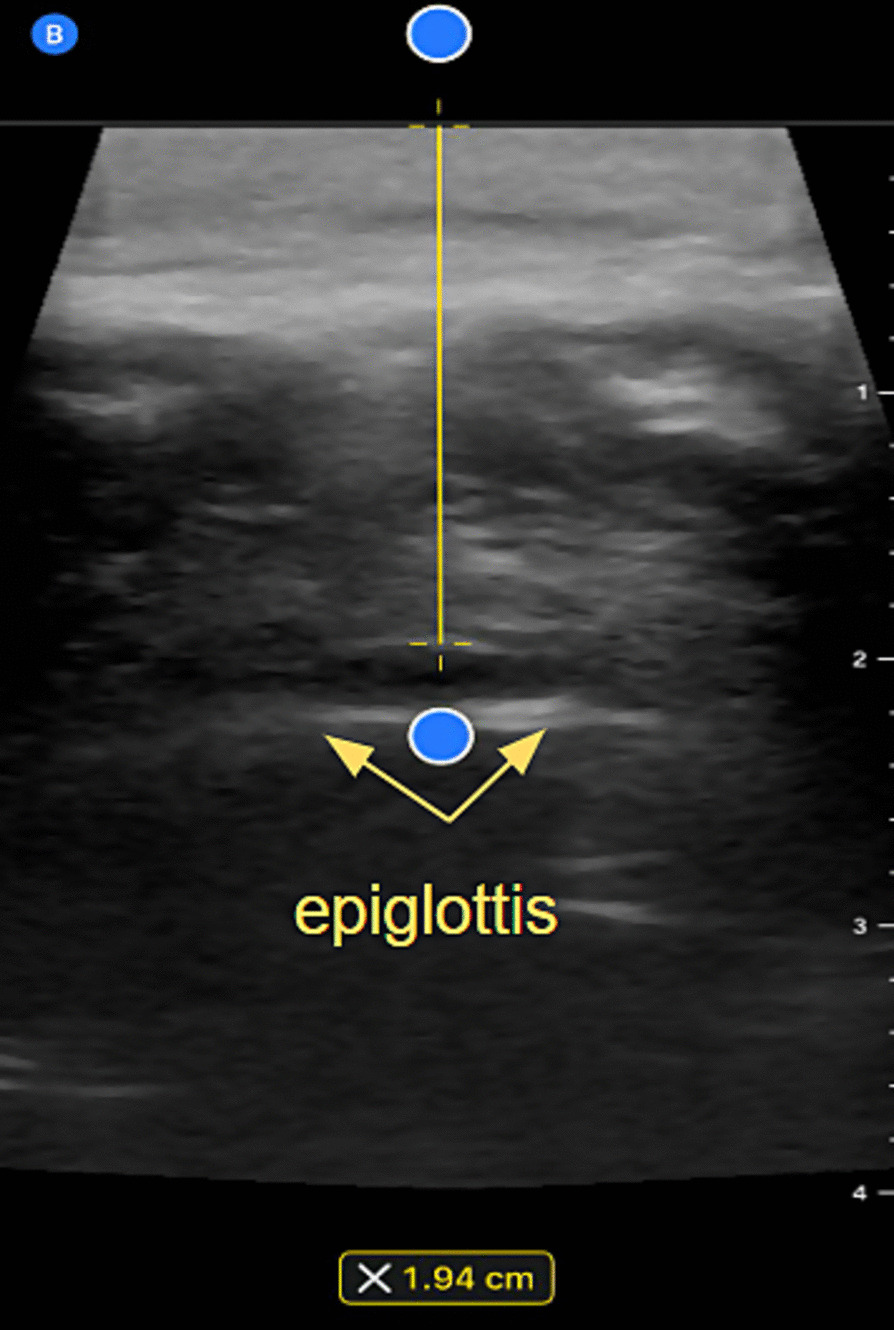
Airway assessment. **A** Upper Lip Bite Test. **B** Modified Mallampati Test. **C **Ultrasound assessment of skin-to-epiglottis distance

A high-frequency linear transducer was used by an experienced anesthetist to measure the skin-to-epiglottis distance (DSE). The operator was not aware of the results of other bedside airway examination tests. With the patient supine and the head in a neutral position, the transducer was placed transversely at the level of the thyrohyoid membrane. The epiglottis was identified as a hypoechoic, curved structure bordered posteriorly by a bright hyperechoic air-mucosa interface [9] (Fig. 1). Three measurements were obtained at the central axis of the epiglottis, and their average was recorded. In this study, we assessed the DSE in the neutral position since it is more common in clinical research [4, 10, 11].

A composite airway score was calculated by assigning one point for each of the following criteria: DSE > 2 cm, ULBT class > I, and MMT grade > II. The total score ranged from 0 to 3. The score was calculated by an independent data collector who was not involved in the airway assessment or intubation process.

In the operating room, standard monitors were applied. Anesthesia was induced with propofol (2 mg/kg), fentanyl (2 µg/kg), atracurium (0.5 mg/kg). Face-mask ventilation was maintained for 3 min. Endotracheal intubation was performed with the head in the sniffing position using an appropriately sized Macintosh laryngoscope blade. The correct placement of the endotracheal tube was confirmed by capnography. External laryngeal manipulation was applied only when deemed necessary by the operating anesthesiologist to improve the laryngeal view.

Cormack–Lehane classification for laryngoscopic view [12] was assessed by the intubating anesthetist as follows: grade 1: most of the vocal cords are seen; grade 2: only the posterior part of the vocal cords or the Arytenoid cartilages are seen; grade 3: only the Epiglottis is seen; grade 4: Epiglottis is not seen. Cormack–Lehane grade > 2 was considered difficult laryngoscopy.

The Intubation Difficulty Scale [13] was also recorded and a score > 5 was considered difficult intubation. Failed intubation was defined as failure of insertion of the endotracheal tube after 2 attempts by a senior anesthetist and the use of alternative devices (e.g., supraglottic device).

### Primary outcome

The ability of the composite airway score to predict difficult laryngoscopy.

### Secondary outcomes

The ability of the composite airway score to predict difficult intubation as well as the ability of each of DSE > 2 cm, ULBT class > I, and MMT grade > II in predicting difficult laryngoscopy and difficult intubation. Other predictors for difficult laryngoscopy and difficult intubation were also studied such as age, sex, body mass index, ASA-PS, mouth opening, thyromental distance, and sternomental distance.

### Statistical analysis

The sample size was calculated using MedCalc software version 14 (MedCalc software bvba, Ostend, Belgium). Assuming a 10% incidence of difficult laryngoscopy [2], a total of 250 patients (with at least 25 positive cases) were required to detect an area under receiver operating characteristic curve (AUC) of 0.7 versus a null hypothesis of 0.5. The study power was set at 90% and alpha error at 0.05.

Data analysis was performed using SPSS version 26 (IBM Corp., NY, USA) and MedCalc. Categorical data were presented as frequencies (%). Patients were categorized according to the difficulty of laryngoscopy and intubation, each classified as either easy or difficult. Continuous data were checked for normality using the Shapiro–Wilk test. Normally distributed continuous data were presented as means ± standard deviations, and skewed data were presented as medians (quartiles). The AUC was calculated for the composite airway score, DSE > 2 cm, MMT > II, and ULBT > I. Youden's index was used to determine optimal cut-off values. Positive predictive value, negative predictive value, and best cut-off points were reported for all predictors. Comparison between different predictors’ AUC was conducted using the DeLong test. Internal validation using bootstrap resampling (1000 iterations) was performed using R software (4.4.1 version). Univariate analysis was performed for determination of the odds ratios (95% confidence intervals [CI]) for each risk factor for difficult laryngoscopy and difficult intubation. The intraclass correlation coefficient (2-way model) was performed to assess agreement among three DSE measurements from each patient. *P*-value < 0.05 was considered statistically significant.

## Results

**Fig. 2:**
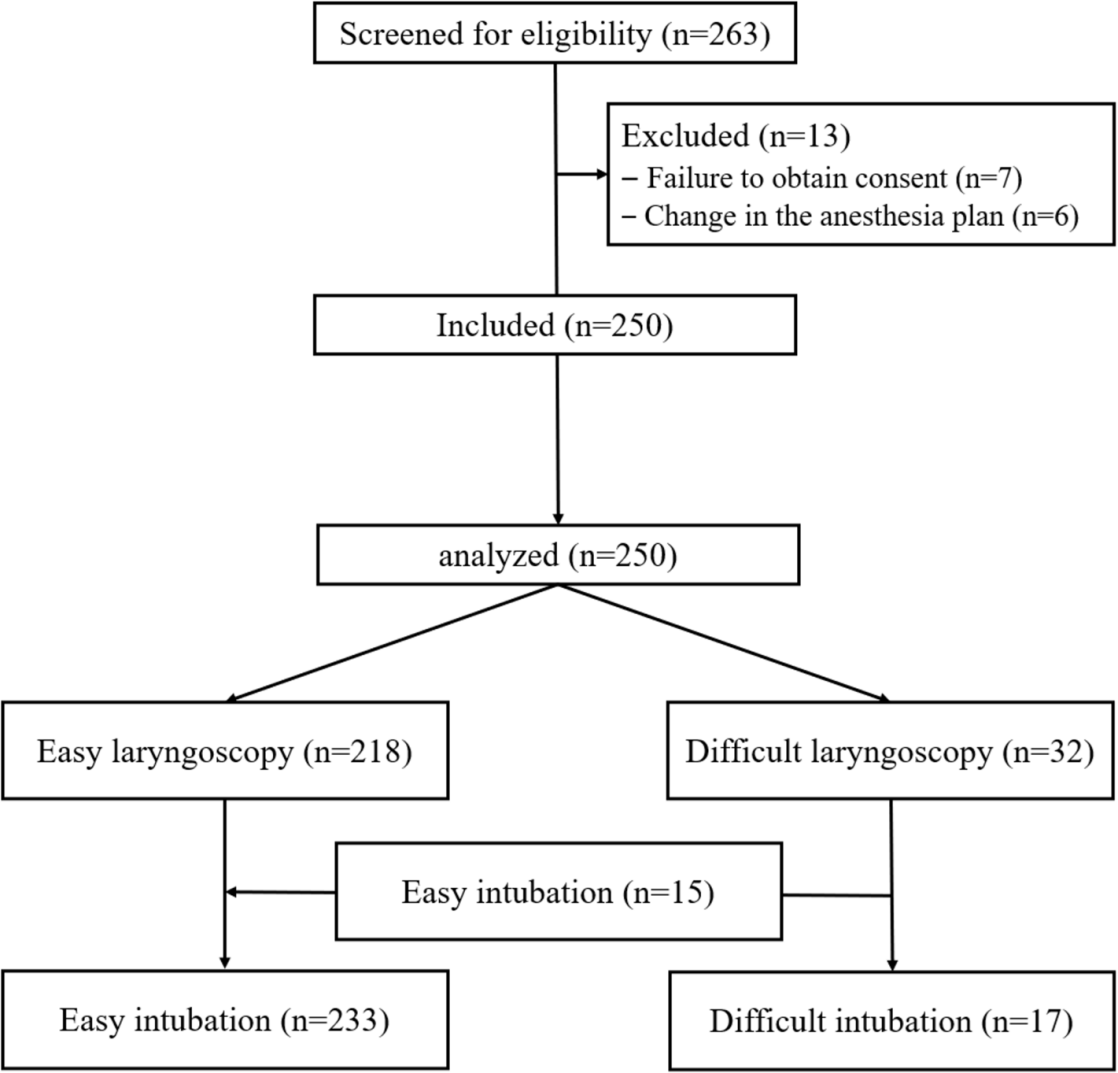
Patients’ enrollment flowchart

In this study, 263 consecutive patients were screened for eligibility; 13 patients were excluded for not meeting the study’s inclusion criteria, and 250 patients were included and were available for the final analysis. The incidence of difficult laryngoscopy and intubation was 12.8% (32/250) and 6.8% (17/250), respectively. None of the patients had failed intubation (Fig. 2).

Patients with difficult laryngoscopy and intubation were older and mostly males, and had higher MMT and ULBT grades, higher DSE, and consequently, higher composite airway score. A composite score of zero was observed in only 2 patients (6.3%) with difficult laryngoscopy and in none of the patients with difficult intubation (Tables 1 and 2).

**Table 1:** Demographic data and airway characteristics according to difficult laryngoscopy

	Easy laryngoscopy (*n* = 218)	Difficult laryngoscopy (*n* = 32)	Odds ratio (95% CI)	*P*-value
Age (years)	45 (42, 51)	52 (45,58)	1.07 (1.02–1.11)	0.005
Weight (kg)	74 ± 14	80 ± 13	1.03 (1.01–1.06)	0.019
BMI (kg/m^2^)	26 (24, 29)	27 (25, 30)	1.08 (0.97–1.20)	0.154
Male sex	130 (59.6%)	26 (81.3%)	2.93 (1.16–7.42)	0.023
ASA-PS I II III	152 (69.7%) 62 (28.4%) 4 (1.8%)	17 (53.1%) 15 (46.9%) 0 (0%)	1.70 (0.86–3.34)	0.127
Thyromental distance (cm)	8.0 (6.5, 9.0)	7.3 (5.6, 8.5)	0.87 (0.69–1.10)	0.255
Sternomental distance (cm)	12.5 (11.5, 14.0)	12.3 (11.6, 14.0)	0.94 (0.77–1.15)	0.568
Mouth opening (cm)	5.0 (4.5, 6.0)	5.0 (4.5, 5.0)	0.84 (0.55–1.27)	0.402
MMT I II III IV	93 (42.7%) 116 (53.2%) 9 (4.1%) 0 (0%)	3 (9.4%) 18 (56.3%) 11 (34.4%) 0 (0%)	6.49 (3.10–13.59)	< 0.001
ULBT Class I Class II Class III	124 (56.9%) 85 (39.0%) 9 (4.1%)	8 (25.0%) 21 (65.6%) 3 (9.4%)	2.73 (1.48–5.06)	0.001
DSE (cm)	1.8 (1.7, 2.1)	2.2 (1.8, 2.4)	1.22 (1.09–1.36)	< 0.001
DSE > 2.0 cm	68 (31.2%)	21 (65.6%)	4.21 (1.92–9.22)	< 0.001
Composite airway score 0 1 2 3	90 (41.3%) 89 (40.8%) 35 (16.1%) 4 (1.8%)	2 (6.3%) 12 (37.5%) 10 (31.3%) 8 (25.0%)	3.63 (2.25–5.86)	< 0.001

Data presented as mean ± standard deviation, median (quartiles), and frequency (%)

*ASA-PS* American Society of Anesthesiologists-Physical Status, *BMI* body mass index, *CI* confidence interval, *DSE* skin-to-epiglottis distance, *MMT* modified Mallampati test, *ULBT* upper lip bite test

**Table 2:** Demographic data and airway characteristics according to difficult intubation

	Easy intubation (*n* = 233)	Difficult intubation (*n* = 17)	Odds ratio (95% CI)	*P*-value
Age (years)	46 (42, 51)	52 (46,59)	1.07 (1.01–1.13)	0.014
Weight (kg)	73 ± 14	81 ± 14	1.03 (0.99–1.07)	0.068
BMI (kg.m^−2^)	26 (24, 29)	27 (25, 29)	1.01 (0.88–1.16)	0.885
Male sex	140 (60.1%)	16 (94.1)	10.63 (1.39–81.51)	0.023
ASA-PS I II III	158 (67.8%) 71 (30.5%) 4 (1.7%)	11 (64.7%) 6 (35.3%) 0 (0%)	1.05 (0.40–2.76)	0.913
Thyromental distance (cm)	8.0 (6.5, 8.9)	7.5 (5.5, 9.3)	0.97 (0.71–1.32)	0.836
Sternomental distance (cm)	12.5 (11.5, 13.8)	12.3 (11.3, 14.0)	0.99 (0.88–1.14)	0.978
Mouth opening (cm)	5.0 (4.5, 6.0)	5.0 (4.5, 5.5)	1.06 (0.63–1.79)	0.822
MMT I II III IV	94 (40.3%) 125 (53.6%) 14 (6.0%) 0 (0%)	2 (11.8%) 9 (52.9%) 6 (35.3%) 0 (0%)	4.83 (2.03–11.48)	< 0.001
ULBT Class I Class II Class III	129 (55.4%) 93 (39.9%) 11 (4.7%)	3 (17.6%) 13 (76.5%) 1 (5.9%)	2.78 (1.26–6.13)	0.011
DSE (cm)	1.8 (1.7, 2.1)	2.3 (2.2, 2.5)	1.41 (1.20–1.64)	< 0.001
DSE > 2.0 cm	75 (32.2%)	14 (82.4%)	9.83 (2.74–35.25)	< 0.001
Composite airway score 0 1 2 3	92 (39.5%) 96 (41.2%) 38 (16.3%) 7 (3.0%)	0 (0%) 5 (29.4%) 7 (41.2%) 5 (29.4%)	4.57 (2.41–8.66)	< 0.001

Data presented as mean ± standard deviation, median (quartiles), and frequency (%)

*ASA-PS* American Society of Anesthesiologists-Physical Status, *BMI* body mass index, *CI* confidence interval, *DSE* skin-to-epiglottis distance, *MMT* modified Mallampati test, *ULBT* upper lip bite test

The AUC (95% CI) for the composite airway score in predicting difficult laryngoscopy and intubation was 0.77 (0.72–0.82) and 0.83 (0.78–0.88), respectively. The AUC (95% CI) using the bootstrapping method was 0.77 (0.69–0.85) for difficult laryngoscopy and 0.83 (0.75–0.91) for difficult intubation.

The AUC for the composite score was higher than that for each of its separate components, the DSE, MMT, and ULBT for both difficult laryngoscopy and intubation (Table 3). A composite score > 1 demonstrated a high negative predictive value of 93% for difficult laryngoscopy and 97% for difficult intubation (Table 3).

**Table 3:** Accuracy of airway tests in predicting difficult laryngoscopy and difficult intubation

	AUC (95% CI)	Sensitivity (95% CI)	Specificity (95% CI)	PPV (95% CI)	NPV (95% CI) %	Cut-off value
Difficult laryngoscopy (*n* = 32/250)						
DSE > 2.0 cm	0.67 (0.61–0.73)	66 (57–81)	69 (62–75)	24 (15–34)	93 (88–97)	
ULBT class > I	0.66 (0.60–0.72)	75 (57–89)	57 (50–64)	20 (14–29)	94 (88–97)	
MMT grade > II	0.65 (0.59–0.71)	34 (19–53)	96 (92–98)	55 (32–77)	91 (86–94)	
Composite airway score	0.77 (0.72–0.82)*†‡	56 (38–74)	82 (76–87)	32 (20–45)	93 (88–96)	> 1
Difficult intubation (*n* = 17/250)						
DSE > 2.0 cm	0.75 (0.69–0.80)	82 (57–96)	68 (62–74)	16 (9–25)	98 (95–100)	
ULBT class > I	0.69 (0.63–0.75)	82 (57–96)	55 (49–62)	12 (7–19)	98 (94–100)	
MMT grade > II	0.65 (0.58–0.71)	35 (14–62)	94 (90–97)	30 (12–54)	95 (92–98)	
Composite airway score	0.83 (0.78–0.88) †‡	71 (44–90)	81 (75–86)	21 (11–34)	97 (95–100)	> 1

*AUC* area under receiver operating characteristic curve, *CI* confidence interval, *DSE* skin-to-epiglottis distance, *PPV* positive predictive value, *MMT* modified Mallampati test, *NPV* negative predictive value, *ULBT* upper lip bite test

^*^denotes significance in relation to DSE

^†^denotes significance in relation to ULBT

^‡^denotes significance in relation to MMT

The sensitivity, specificity, and predictive values of different cut-off points of the composite score are presented in Table 4.

**Table 4:** Predictive value of different cut-offs of the composite airway score

	Sensitivity (95% CI)%	Specificity (95% CI)%	PPV (95% CI)%	NPV (95% CI)%
Difficult laryngoscopy				
1	94 (79–99)	41 (35–48)	19 (13–26)	98 (92–100)
2 or 3 (i.e., > 1)	56 (38–74)	82 (76–87)	32 (20–45)	93 (88–96)
3	25 (12–43)	98 (95–100)	67 (35–90)	90 (85–93)
Difficult intubation				
1	100 (81–100)	40 (33–46)	11 (6–17)	100 (96–100)
2 or 3 (i.e., > 1)	71 (44–90)	81 (75–86)	21 (11–34)	97 (94–99)
3	29 (10–56)	97 (94–99)	42 (15–72)	95 (91–97)

*CI* confidence interval,* PPV* positive predictive value, *NPV* negative predictive value

The intraclass correlation coefficient for ultrasound measurement of DSE was 0.94 (95% CI 0.93–0.95) for single measures and 0.98 (95% CI 0.97–0.98) for average measures.

## Discussion

In this study, we evaluated the ability of a composite airway score integrating ultrasound examination (DSE) with clinical examination (ULBT and MMT) to predict difficult airway. The composite airway score improved the accuracy of predicting both difficult laryngoscopy and difficult intubation compared to the individual tests alone, with better predictive performance for difficult intubation than for difficult laryngoscopy. This improvement can be attributed to the integration of multiple predictors, each reflecting a distinct anatomical or functional aspect of the airway: the ULBT evaluates mandibular mobility, the MMT assesses oropharyngeal soft tissue volume and mouth opening grade, and the DSE reflects the volume and compliance of the submandibular space. By incorporating these complementary elements, the composite score provides a more comprehensive assessment of airway difficulty. The robustness of these findings was confirmed through internal validation using the bootstrapping method.

At the optimal cut-off value (> 1), the score had a negative predictive value of 93% for difficult laryngoscopy and 97% for difficult intubation. This suggests that if all tests are negative, or only one is positive, the score can reliably exclude a difficult airway in most patients. When all three tests are negative, the composite score has a sensitivity and negative predictive value of 100% for prediction of difficult intubation.

The use of composite scores that combine clinical predictors with ultrasound measurements for airway assessment remains underexplored. Consistent with our findings, Parameswari et al. [6] reported that the inclusion of DSE improved the sensitivity of the MMT. Similarly, Daggupati et al. [5] demonstrated that adding DSE to a clinical scoring system—which included mentohyoid distance, mandibular subluxation, and head extension—enhanced both the accuracy and sensitivity of clinical assessment. However, these studies lack external validation, as the DSE cut-off values were derived from the same patient cohorts in which they were tested, thereby limiting their generalizability. Our study has several advantages over previous reports: first, we validated the use of DSE with a previously established value of 2.0 cm in our patient population. At the time of preparing this research, the available evidence suggested optimal DSE thresholds ranging between 1.6 and 2.8 cm, with the best predictive performance observed in the 2.0–2.5 cm range [4], which was supported by subsequent metanalyses [10, 11, 14]. Because airway screening tools prioritize sensitivity, we selected a threshold of 2 cm, the lower value of the optimal range.

Second, we used the Intubation Difficulty Scale while previous studies used the Cormack–Lehane classification which has several limitations such as its subjective nature which exhibits poor inter- and intra-rater reliability [15]. Moreover, a poor laryngeal view does not always translate into difficult intubation. For example, in our study, of the 32 patients with difficult laryngoscopy, only 17 were found to have truly difficult intubation based on the Intubation Difficulty Scale. The Intubation Difficulty Scale provides a more objective and standardized assessment and includes several factors which impact the intubation difficulty such as the use of external laryngeal manipulation.

Preoperative airway assessment in persons with no apparent anatomical abnormalities serves primarily as a screening tool to exclude difficult intubation. Therefore, high sensitivity is essential as the ultimate goal of screening is capturing all positives (i.e., high negative predictive value). However, most currently available clinical airway assessments are characterized by high specificity but low sensitivity, and this impairs their screening ability [2, 16].

Ultrasound assessment of the airway has gained increasing attention due to its ability to visualize the sub-hyoid and deeper soft tissue structures, potentially identifying features associated with difficult intubation that are not apparent on routine clinical examination [4]. Among various ultrasound-based predictors, the DSE has shown the highest diagnostic accuracy, particularly due to its relatively high sensitivity in detecting difficult airways [4].

Since no single test perfectly predicts difficult airway, current guidelines recommend using a combination of validated tests [1]. Our findings demonstrate that the composite airway score improved the overall accuracy for predicting difficult airway, with particularly strong performance as a screening tool for ruling out difficulty, especially when all score components are negative. However, the relatively low positive predictive values, attributable to the low prevalence of difficult laryngoscopy in our cohort, limit the score’s confirmatory ability.

This study had some limitations. It was conducted in a single center; however, it was a large tertiary center that covers a large geographic area. Regarding the cut-off value for DSE, we relied on a value supported by previous literature and selected a threshold that could favor diagnostic sensitivity in addition to being memorable. Future studies are needed to refine the model including additional predictors such as age and male sex; and assessment of other acceptable cut-off values for the DSE. We did not include patients with suspected difficult airway as the aim of this study was to identify patients at risk of difficult airway among those with apparently normal airway. We did not include patients with obesity because the cut-off value used in the study was derived from populations with a BMI mostly below 35 kg/m^2^. Therefore, future studies are needed to confirm our findings in other populations, including high-risk patients and those with high BMI. In this study, we used the original Cormack–Lehane classification to assess the laryngoscopic view rather than the modified Cormack–Lehane classification [17], which may offer greater discriminatory precision, particularly in distinguishing Grade 2 views into 2a and 2b. The original classification was chosen to maintain comparability with the majority of existing literature on difficult airway prediction [2]. Nonetheless, we additionally employed the Intubation Difficulty Scale score [13], a composite, multi-dimensional measure that captures the entire intubation encounter beyond laryngoscopic view alone, which partially mitigates this limitation. The Cormack–Lehane grade was recorded based on the first encountered laryngoscopic view, and external laryngeal manipulation was applied only when deemed necessary by the operating anesthesiologist to optimize the laryngeal view. This approach may have resulted in overestimation of laryngoscopic difficulty. However, this limitation is partially mitigated by our use of the Intubation Difficulty Scale, which incorporates external laryngeal manipulation as one of its components, thereby providing a more objective assessment of intubation difficulty. We did not assess the depth of neuromuscular blockade prior to laryngoscopy. However, laryngoscopy was performed 3 min after the administration of atracurium, which is an acceptable time to reach its peak neuromuscular blocking effect at the dose used [18]. This approach is consistent with standard clinical practice and has been widely adopted in the literature [19]

## Conclusion

A composite airway score that combines an ultrasound-derived parameter (DSE) with clinical predictors (ULBT and MMT) can accurately predict difficult laryngoscopy and difficult intubation. When all three parameters are negative (score equals zero), difficult intubation is very unlikely. Additionally, when the score is less than 2, difficult laryngoscopy and difficult intubation are ruled out with accuracy of 93% and 97%.

This manuscript follows the STROBE guideline for reporting observational studies.

## Data Availability

The datasets used during the current study are available from the corresponding author on reasonable request.
